# Unbiased estimation of odds ratios: combining genomewide association scans with replication studies

**DOI:** 10.1002/gepi.20394

**Published:** 2009-01-12

**Authors:** Jack Bowden, Frank Dudbridge

**Affiliations:** ^1^MRC Biostatistics Unit, Institute of Public HealthCambridge, UK

**Keywords:** genomewide scans, winner's curse, selection bias, UMVUE, WTCCC

## Abstract

Odds ratios or other effect sizes estimated from genome scans are upwardly biased, because only the top-ranking associations are reported, and moreover only if they reach a defined level of significance. No unbiased estimate exists based on data selected in this fashion, but replication studies are routinely performed that allow unbiased estimation of the effect sizes. Estimation based on replication data alone is inefficient in the sense that the initial scan could, in principle, contribute information on the effect size. We propose an unbiased estimator combining information from both the initial scan and the replication study, which is more efficient than that based just on the replication. Specifically, we adjust the standard combined estimate to allow for selection by rank and significance in the initial scan. Our approach explicitly allows for multiple associations arising from a scan, and is robust to mis-specification of a significance threshold. We require replication data to be available but argue that, in most applications, estimates of effect sizes are only useful when associations have been replicated. We illustrate our approach on some recently completed scans and explore its efficiency by simulation. *Genet. Epidemiol*. 33:406–418, 2009. © 2009 Wiley-Liss, Inc.

## INTRODUCTION

Genomewide scans are becoming increasingly popular as a tool for estimating the association between large numbers of genetic variants, usually single-nucleotide polymorphisms (SNPs), and many common, complex diseases [Hirschhorn and Daly, 2005]. Although large effects are not expected for any single SNP, the hope is that a set of SNPs can be identified as explaining a sizable proportion of disease risk. Statistical analyses of genome scans are mainly focused on hypothesis testing, with estimation of the corresponding effect sizes regarded as a secondary goal. This is because of the exploratory nature of a scan: we are looking to identify associated markers from a set of anonymous SNPs, whereas estimation is typically required for a known risk factor. Nevertheless, knowledge of the effect sizes of the SNPs found in a scan can be useful, for example in designing replication studies with appropriate sample sizes [Yu et al., 2007]. Applications such as estimating the proportion of heritability explained [Easton et al., 2007], performing meta-analyses [Lohmueller et al., 2003], and constructing risk profiles [Janssens et al., 2008] are usually concerned with confirmed, replicated associations; but if the relevant markers were initially found in a scan, then it would contain information that is useful in those situations too. Such applications are likely to assume greater importance with time, as methodology improves to ensure multiple true associations in most genome scans.

It is well known that naïve estimates of odds ratios or other effect sizes from a genome scan are upwardly biased. This problem was introduced to the human genetics literature by Göring et al. [2001] in the context of linkage analysis, and was further elucidated for genomewide association scans by Garner [2007]. We want to distinguish two sources of the upward bias. Significance bias arises when estimation is performed only for effects that are statistically significant: the expected value of an estimator, conditional on it being significant, is then typically higher than its unconditional expectation, which for an unbiased estimator is the population value. This is the same principle that underlies publication bias in scientific literature [Hedges, 1992; Copas, 1999]. Ranking bias arises when the quantities estimated depend upon a rank ordering related to those same quantities. Thus, if following a genome scan SNPs are ranked by their *P*-values, then the expected value of an estimator, conditional it being the most significant, is again greater than its unconditional expectation. Ranking bias applies not only to the most significant SNP, but also to the second most significant, third most and so on, and is present even when there is no selection by significance. This is similar to the “winner's curse” effect [Thaler, 1988], in its original sense that the winner of a common value auction tends to overpay; the difference here is that SNPs do not have a common effect size, but the bias will operate to some extent whenever there is incomplete information on the effect size. A similar phenomenon also arises in clinical trials when multiple outcomes have been measured but only the most extreme outcome is reported [Hutton and Williamson, 2000; Williamson et al., 2005].

Although unbiased estimates of SNP effects are available from replication studies [Göring et al., 2001], it would be desirable to obtain them from genome scan data since their sample sizes are typically large and should therefore contain useful information. Several authors have, therefore, proposed to infer bias corrected estimates based solely on the initial sample data. To address significance bias Zöllner and Pritchard [2007] proposed to maximize the likelihood of the genotype data conditional on it passing a significance threshold. Using a general notation their approach seeks to maximize 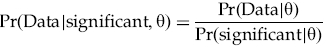
(1) over parameters θ, here the odds ratio and allele frequency. They applied this approach to multinomial data from contingency tables. Ghosh et al. [2008] and Zhong and Prenctice [2008] applied the same principle to the summary odds ratio, assumed to be normally distributed with its variance equal to the sample estimate. Although this is, in a sense, the correct model for the data, it does not lead to an unbiased estimate for θ, as noted by those authors, and their results suggest a tendency to overcorrect.

Ranking bias was addressed by Sun and Bull [2005], who used bootstrapping and cross-validation to correct for the bias of point estimates for the top-ranking SNP. Their work is mainly developed for linkage analysis, and would seem to be very time-consuming for association analysis on the genomewide scale, although an application to a candidate-gene screen has been reported [Yu et al., 2007]. Again, this approach resulted in reduction of bias without achieving unbiased estimation.

In fact, while these methods show that bias corrections based on heavily selected data can be made, it is known that there is no unbiased estimate for the top-ranking effect [Putter and Rubinstein, 1968; Stallard et al., 2008], suggesting that the search for general unbiased estimators is futile. Here we take a different approach, by assuming that a replication study has been performed and then combining the estimates from both the initial scan and the replication to obtain an unbiased estimate that is more efficient than that based only on the replication data. In particular, our estimator has minimum variance, in a certain sense, among all unbiased estimators that use the full complement of data. We account for both significance bias and ranking bias, and our approach explicitly allows and adjusts for multiple associations in a scan. Although we need replication data to be available, we feel that this is not overly restrictive, because most applications of estimation will be concerned with validated associations. Indeed, replication is accepted as a sine qua non of genome scans [Chanock et al., 2007], so it is debatable whether we should be much concerned with estimation from the scan data only. Of course, the design of a replication study requires some estimate of effect sizes, but since replication will typically be attempted for several associations concurrently, a rough indication of the order of magnitude would suffice and this can be obtained by existing methods.

Our methodology originates from the adaptive clinical trial literature, and we will draw some comparisons between the two fields. In the “Methods” section we describe our estimation procedure together with measures of bias and mean square error that are appropriate in this context. In the section “Results” we apply our approach to studies in the Wellcome Trust Case Control Consortium [WTCCC, 2007], leading us to illustrate some important aspects through simulations. We conclude with a “Discussion”.

## METHODS

### TWO-STAGE UNBIASED ESTIMATION

We assume a design consisting of a genomewide association scan (stage 1) followed by a replication study (stage 2) in which only SNPs meeting some selection criteria in stage 1 are of interest. Let *X* = {*X*_*i*_, *i* = 1,…, *k*} be the estimated effect sizes for *k* SNPs in stage 1, assumed to follow independent 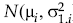 distributions. In the case-control design *X_i_* will usually be log odds ratios. μ_*i*_ is unknown but its variance, although estimated in practice, will be assumed known. Denote the ordered stage 1 effects as *X*_(*i*)_ and define μ_(*i*)_ = μ_*j*_ when *X_(*i*)_* = *X_j_*. Note that we define μ_(*i*)_ as the mean of the *i*th ranked effect, not the *i*th ranked mean, and that therefore μ_(*i*)_ is a function of *X*. Define σ_1,(*i*)_ similarly. Let ϒ*_i_* be the stage 2 estimate of the *i*th ranked effect in stage 1, so ϒ_*i*_ 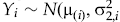. The problem is to estimate μ_(*i*)_ subject to selection criteria including specified values of *i* and a *P*-value threshold.

This scenario is similar to the pharmaceutical setting, particularly in early experimental trials, in which one is faced with determining the most promising of several experimental treatments. A well established and cost-effective strategy is to test all *k* treatments in a first stage and to select the best performing treatment with the largest efficacy score, *X*_(1)_, for further investigation [Thall et al., 1988; Stallard and Todd, 2003; Sampson and Sill, 2005]. When tested in isolation in stage 2, the efficacy score of this best performing stage 1 treatment, ϒ_1_, follows 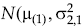. Since it is the maximum of *k* normally distributed random variables *E*[*X*_(1)_] ≠ μ_(1)_ [for details on the exact distribution of *X*_(1)_ see Sun and Bull, 2005]. The second-stage data give an unbiased estimate of μ_(1)_, but it is often overlooked on account of its large variance. For this reason a weighted average of the first- and secondstage data is sometimes taken, giving an estimator of the form

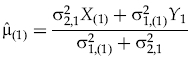
(2)
which can be regarded as a maximum likelihood estimator (MLE). However, 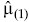 is biased because no account is made for selection at stage 1. For all stage 1 and 2 variances equal Cohen and Sackrowitz [1989] proposed an unbiased estimate for μ_(1)_ of the form

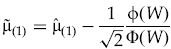
(3)
where φ(.) and Φ(.) are the pdf and cdf of the standard normal distribution and 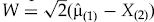. This can be seen as a correction to the MLE which depends on *X*_(2)_, the second best performing treatment in stage 1. The two-stage estimation procedure is necessitated by the fact that no unbiased estimate for μ_(1)_ exists based only on (finite) stage 1 data—see Stallard et al. [2008] for a theoretical explanation. This explains why the “bias corrected” estimates of Zöllner and Pritchard [2007], Ghosh et al. [2008], and Zhong and Prenctice [2008] are, nevertheless, still biased. In recent work Bowden and Glimm [2008] generalized this result to the *i*th ranked stage 1 effect, and also to allow unequal variances among all stage 1 and stage 2 estimates. The estimate for μ_(*i*)_ in this case is

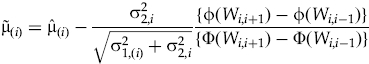
(4)
where

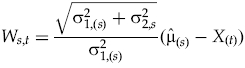
(5)
The MLE for the *i*th ranked effect is corrected by taking into account information from the (*i* – 1)th and (*i* + 1)th ranked stage 1 effects. In this formula *X*_(0)_ and *X*_(*k*+1)_ are defined as ∞ and –∞, respectively. The full proof of this result can be found in Bowden and Glimm [2008] but to motivate subsequent results we provide a summary of the main steps.

Without loss of generality we assume that the event *Q*: *X*_1_ ≥ *X*_2_ ≥ … ≥ *X_k_* has occurred, so that *X_i_* = *X_(i)_*. Let 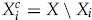. The pair 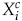 and *Z_i_* = (σ_2,*i*_/σ_1,*i*_)*X*_*i*_)+(σ_1,*i*_/σ_2,*i*_)ϒ_*i*_ are then sufficient and complete statistics for μ_1_,…, μ_*k*_. The joint distribution of ϒ_*i*_ and *X*_1_,…,*X_k_* given *Q*, *f*(ϒ*_i_*,*X*|*Q*) is then transformed into *f*(*X*, *Z_i_*|*Q*) and 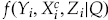. The joint density 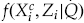 is obtained from the integral


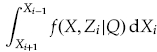
(6)

which enables the density 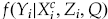 to be expressed as the ratio 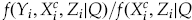. This is greatly simplified due to numerous cancellations, in particular the selection probabilities which are analogous to those that feature in the denominator of, and cause problems for, formula (1). Using the Rao-Blackwell theorem formula (4), which is 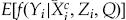, is the uniformly minimum variance unbiased estimator for μ_(*i*)′_, conditional on Q (we call it the UMVCUE). This means that, given the ranking in stage 1, the estimator is unbiased for the corresponding effects and has minimum variance among all such unbiased estimators.

We now propose some modifications to this formula in order to apply the same estimation procedure to a genome scan followed by replication. Instead of the magnitude of the point estimates determining the rank order, it is more common in the genomewide setting to rank SNPs according to the statistical significance of their effects, and to restrict attention to those passing an initial *P*-value threshold *p_crit_*. For a one-sided Wald-type test, we can make this extension by conditioning instead on the event


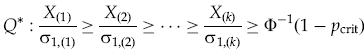
(7)

This leads to slight changes in the proof since conditioning on Q^*^ as opposed to *Q* changes the limits of integration for *X_i_* and *Y_i_*, with the result that *W_s,t_* in (4) becomes


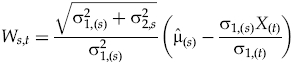
(8)

For (8) to work generally we define *X*_(*k*+1)_/σ_1,(*k*+1)_ = Φ^−1^(1 – *p*_crit_). This expression was noted in Bowden and Glimm [2008], though they did not allow for a *P*-value threshold. If SNPs that confer either an increased or decreased disease risk are of equal interest, as is usually the case, then the rank order of significance should be based on two-sided *P*-values. This now requires conditioning on the event


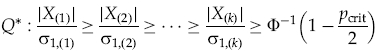
(9)

In Appendix A we show that the UMVCUE now becomes


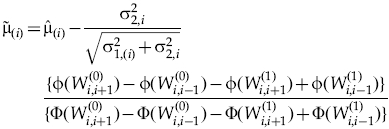
(10)

where


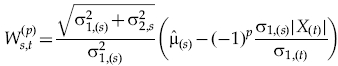
(11)

and |*X*_(*k*+1)_|/σ_1,(*k*+1)_=Φ^−1^(1 – *p*_crit_/2).

We shall work with this last form in the remainder of the article. Note that this estimator may be easily calculated in standard software, using only the summary estimates obtained in the two stages.

### ASSESSING POINT ESTIMATOR PERFORMANCE

We have proposed an unbiased estimate 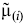 for the effect size of a SNP association, conditional on its rank order and its passing a *P*-value threshold. Our intention is to compare the usefulness of this estimate to alternatives based on two-stage data. To do this we will calculate the bias and mean squared error of each estimator, to assess their bias-variance trade-offs. However, a subtle facet of this estimation problem is that the true effect of the *i*th ranked SNP, μ_(*i*)_ is not a fixed parameter, but is a random variable that can take any of the values μ_1_,…,μ_*k*_. This means that standard formulas, such as that for the standard error of the MLE, are inaccurate because they do not allow for the variability in the effect being estimated. Furthermore, no exact formula for the UMVCUE's variance is known [Cohen and Sackrowitz, 1989; Sill and Sampson, 2007]. Therefore, in accordance with Posch et al. [2005], Sill and Sampson [2007], and Bowden and Glimm [2008] we choose to evaluate a generic estimator 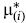 with the quantities


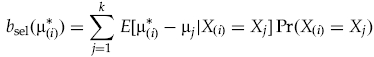
(12)


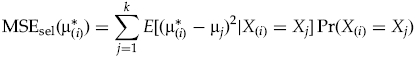
(13)

These expressions are the weighted average bias and mean squared error over the distribution of the random variable μ_(*i*)_, which takes the value μ_*i*_ with probability Pr(*X*_(*i*)_ = *X_i_*) and can be evaluated to a high degree of accuracy by Monte-Carlo simulation.

This point of view also holds that the usual formula for the standard error of the stage 2 estimate is inaccurate when applied to estimating the top-ranking effects in stage 1. If a fixed SNP is specified in advance, then the usual standard error does reflect the sampling variation in stage 2, but if the SNP is regarded as random then the weighted average (13) is more appropriate. This forces a distinction between whether a SNP is regarded as definitively associated after stage 1, in which case the fixed effect view is reasonable, or whether stage 2 is regarded as an integral part of the discovery process, suggesting the random effect view. Both views are justifiable in real contexts, but our view is that if we will combine estimates from both stages, then we should allow for variation across both stages even when basing an estimate on stage 2 data only. Although this distinction may seem technical, it will be important for illustrating our claim that the UMVCUE estimator is more efficient than that based only on the stage 2 data.

## RESULTS

### APPLICATION TO WTCCC DATA

In 2007, the Welcome Trust Case Control Consortium published genome scans of seven common diseases [WTCCC, 2007]. About 2,000 cases for each disease and a common set of 3,000 controls were genotyped. While intended as a proof of concept, the study was perhaps more successful than expected, as for each disease a number of associations were successfully replicated. We illustrate our estimation approach on the results for two diseases: type-1 diabetes (T1D) [Todd et al., 2007] and Crohn's disease (CD) [Parkes et al., 2007]. These two were chosen because replication was attempted for several SNPs (10 and 11, respectively) and because both articles reported summaries of both the WTCCC and the replication data from which odds ratios and standard errors could be recovered. The two studies differed in the size of the replication cohort, which has implications for mean squared error. Our results are intended to be illustrative and should not be taken as definitive estimates, as we have made some assumptions that may not have held in the original studies.

Todd et al. [2007] report robust associations for 15 SNPs in the WTCCC data, four of which were previously known and for which replication was not attempted. One SNP was identified by a 2-df genotype test, which we excluded because it is unclear how to allow for selection by the genotype test when estimating the allelic odds ratio. A follow-up study of 4,000 cases and 5,000 controls attained nominal significance for allelic tests of 7 SNPs out of 10 attempted. In Table I we show the estimated odds ratios from the WTCCC data and replication data, followed by three estimates that combine the two stages: the ordinary MLE, the corrected MLE of Zhong and Prenctice [2008] (called ZP), and our UMVCUE. For the ZP estimator we used the weighted average of corrected and uncorrected estimators (their equation 3.4) using the maximum likelihood adjusted estimator for a two-stage design with selection after the first stage only (their equation 2.3). For the ZP estimator and our UMVCUE we assumed a *P*-value threshold of 2.25 × 10^−5^, which is the *P*-value for the Wald test of the least significant SNP taken forward. This was certainly not the selection criterion used in the original study, but it suffices for our illustration.

**TABLE I:** The odds ratios (95% CIs) of 10 SNPs reported by Todd et al. [2007] in an initial scan (stage 1) and replication study (stage 2)

Chr	SNP	Stage 1 OR	Stage 2 OR	GZW	MLE	ZP	UMVCUE
12q24	rs17696736	1.37 (1.27, 1.48)	1.16 (1.09, 1.24)	1.37	1.23	1.23	1.19
12q13	rs2292239	1.30 (1.2, 1.41)	1.28 (1.20, 1.37)	1.29	1.29	1.29	1.27
16p13	rs12708716	1.30 (1.19, 1.42)	1.20 (1.13, 1.29)	1.27	1.24	1.23	1.21
18p11	rs2542151	1.33 (1.2, 1.48)	1.29 (1.20, 1.38)	1.27	1.30	1.30	1.29
4q27	rs17388568	1.27 (1.15, 1.40)	1.08 (1.01, 1.16)	1.20	1.14	1.10	1.08
5q14	rs7722135	1.27 (1.15, 1.40)	1.09 (1.01, 1.17)	1.11	1.15	1.10	1.08
2q11	rs9653442	1.21 (1.11, 1.31)	1.07 (1.00, 1.15)	1.08	1.10	1.06	1.05
2q13	rs6546909	1.31 (1.16, 1.47)	1.02 (0.95, 1.10)	1.09	1.12	1.04	1.02
10p11	rs2666236	1.21 (1.11, 1.31)	1.05 (0.98, 1.13)	1.05	1.11	1.08	1.07
1q32	rs12061474	1.33 (1.18, 1.54)	1.00 (0.93, 1.08)	1.08	1.10	1.04	1.00

Columns 1–4: Odds ratios are shown in the direction of increased risk. SNPs are ranked in order of stage 1 statistical significance. Columns 5–8: The one-stage correction of Ghosh et al. [2008] (GZW), the two-stage MLE, two-stage corrected MLE of Zhong and Prenctice [2008] (ZP), and our UMVCUE for the *i*th ranked stage 1 SNP's true odds ratio. MLE, maximum likelihood estimator; SNP, single-nucleotide polymorphism.

The stage 1 estimate is consistently higher than the stage 2 estimate and the UMVCUE, reflecting the upward bias. The MLE is similar to the combined odds ratios reported by Todd et al. [2007], and also shows an upward bias. The ZP estimate is consistently less than the MLE but greater than the UMVCUE, suggesting an incomplete bias correction. Since simulations based on only one significant SNP indicated a tendency to overcorrection [Zöllner and Pritchard, 2007; Ghosh et al., 2008], the upward bias here is likely due to uncorrected ranking bias. The UMVCUE is in good agreement with the stage 2 estimate, but can be higher or lower after taking the stage 1 results into account.

Parkes et al. [2007] report successful replication of 12 SNPs in a follow-up cohort of 1,182 cases and 2,024 controls. Of these, the significance of one (rs6887695) in the WTCCC scan was severely attenuated after data cleaning (*P* = 0.01) so we exclude this from our illustration. In Table II we show the same five estimators of the odds ratios. The *P*-value threshold was now taken at 4.9 × 10^−5^. The general pattern is the same in that the MLE and ZP show bias, whereas the UMVCUE is similar to the stage 2 estimate. In two cases, rs4958847 and rs10801047, the stage 2 estimates are actually higher than stage 1, which reflects sampling variation. The UMVCUE allows for this whereas for rs10801047 ZP has adjusted the MLE back toward the stage 1 estimate. Also the UMVCUE for the 2nd ranking SNP rs9292777 is higher than both the stage 1 and stage 2 estimates. This reflects a negative correction to the MLE, occurring because the stage 1 estimate shows less bias than expected given its ranking.

**TABLE II:** The odds ratios (95% CIs) of 11 SNPs estimated from allele frequencies reported by Parkes et al. [2007] in an initial scan (stage 1) and replication study (stage 2)

Chr	SNP	Stage 1 OR	Stage 2 OR	GZW	MLE	ZP	UMVCUE
5p13	rs17234657	1.55 (1.38, 1.74)	1.16 (1.05, 1.29)	1.55	1.39	1.39	1.16
5p13	rs9292777	1.38 (1.26, 1.51)	1.34 (1.21, 1.49)	1.38	1.37	1.37	1.39
10q24	rs10883365	1.27 (1.17, 1.38)	1.18 (1.02, 1.37)	1.26	1.24	1.24	1.16
18p11	rs2542151	1.35 (1.21, 1.50)	1.15 (1.00, 1.32)	1.32	1.27	1.25	1.15
5q33	rs13361189	1.51 (1.30, 1.76)	1.38 (1.20, 1.59)	1.46	1.46	1.45	1.40
3p21	rs9858542	1.26 (1.15, 1.38)	1.17 (1.02, 1.34)	1.21	1.22	1.21	1.17
5q33	rs4958847	1.35 (1.20, 1.53)	1.36 (1.19, 1.56)	1.26	1.36	1.35	1.35
5q23	rs10077785	1.29 (1.16, 1.43)	1.19 (1.04, 1.36)	1.20	1.25	1.22	1.19
1q24	rs12035082	1.22 (1.12, 1.33)	1.14 (0.99, 1.31)	1.15	1.19	1.17	1.15
21q22	rs2836754	1.22 (1.12, 1.33)	1.15 (1.01, 1.32)	1.11	1.19	1.16	1.16
1q31	rs10801047	1.38 (1.18, 1.61)	1.47 (1.29, 1.68)	1.09	1.42	1.39	1.44

Columns 1–4: SNPs are ranked in order of stage 1 statistical significance. Columns 5–8: The one-stage correction of Ghosh et al. [2008] (GZW), the two-stage MLE, corrected MLE of Zhong and Prenctice [2008], and our UMVCUE for the *i*th ranked stage 1 SNP's true odds ratio. MLE, maximum likelihood estimator; SNP, single-nucleotide polymorphism.

For comparison we also report the bias reduced estimates proposed by Ghosh et al. [2008], based only on the stage 1 data (specifically their “compromise estimator” μ3). Of course, if two-stage data are available it would make sense to use the full set of data, but it might be that one stage is sufficient for accurate estimation. The estimates in Tables I and II suggest that the bias correction is less complete than for the two-stage estimators, and the top-ranking SNPs remain severely biased owing to the correction for significance only. The estimates are fairly sensitive to the choice of *P*-value threshold, leading to differences between our results and those of Ghosh et al. [2008]. For example, for rs2542151 in T1D our estimated odds ratio was 1.27 using a *P*-value threshold of 2.25×10^−5^, whereas Ghosh et al. [2008] obtained 1.09 using a *P*-value threshold of 5 × 10^−7^.

### STOCHASTIC NATURE OF THE UMVCUE AND THE CONDITIONAL MLE

**Fig. 1:**
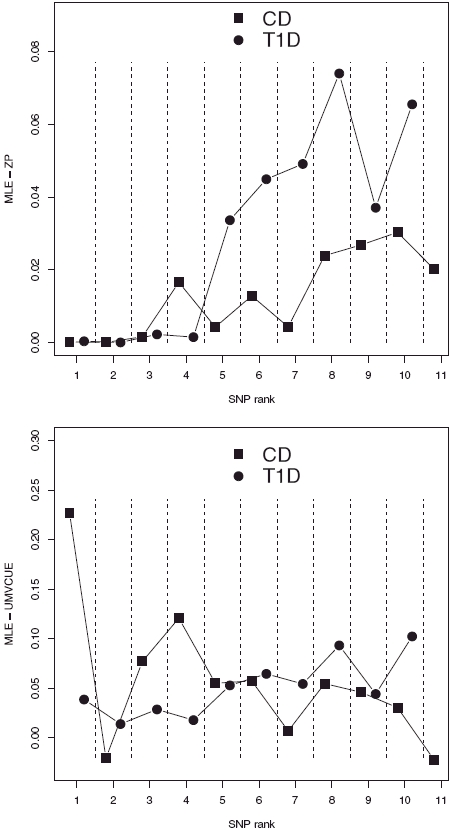
Top: Difference between the MLE and the corrected MLE of Zhong and Prenctice [2008]. Bottom: Difference between the MLE and the UMVCUE. MLE, maximum likelihood estimator.

To gain some insight into the nature of these estimators, we show in Figure 1 the difference between the unadjusted combined log-odds ratios and the two bias adjusted estimators. The ZP estimator, which adjusts the estimates for passing a significance threshold *p*_crit_ at stage 1, makes no correction for the two most significant stage 1 SNPs found by Todd et al. [2007] or Parkes et al. [2007]. This is because these SNPs have *P*-values so far away from *p*_crit_ that selection is judged to have no biasing effect. Conversely, the largest correction is made to the SNPs that are least significant, and therefore closer to *p*_crit_. On the other hand, the adjustments made by our UMVCUE are not only uniformly greater but also take the rank into account, leading to a less obvious relationship between the rank and the degree of adjustment.

**Fig. 2:**
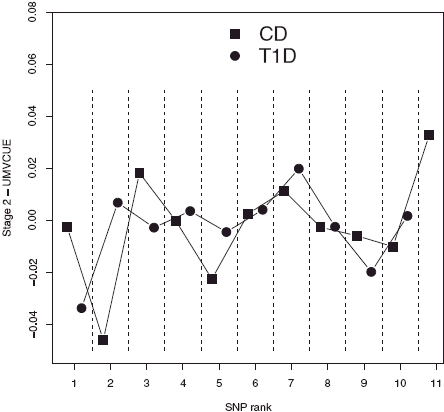
Difference between the stage 2 estimates and the UMVCUE.

Figure 2 plots the difference between the stage 2 estimates and our UMVCUE. We see that the bias corrected UMVCUE is distributed around the unbiased stage 2 estimate. The correlation between the UMVCUE and the stage 2 estimated log-odds ratios is 0.99 and 0.98 for the T1D and CD data, respectively, and the two estimates are very close, differing by less than 0.05. It might seem that the UMVCUE adds little value to the simple stage 2 estimate, but that conclusion would be misleading. In the T1D data, stage 2 is rather larger than stage 1, with the result that the unadjusted MLE is already quite close to the stage 2 estimate, so that the bias adjustment brings the estimate closer still to stage 2. In the CD data, however, the ZP estimate is generally close to the unadjusted MLE, suggesting that the significance bias is not severe, but there is a strong discrepancy with the UMVCUE for the top- and bottom-ranking SNPs. Here, the ranking in stage 2 is so discrepant from that in stage 1 that the conditioning on rank used by the UMVCUE results in much stronger weight being placed on stage 2. So for example, the information on rs17234657 provided by stage 2 suggests that its top ranking in stage 1 is very unlikely, and thus that the stage 1 estimate is severely biased. The closeness of the UMVCUE to the stage 2 estimate in this case is therefore due to the rank adjustment of the UMVCUE and not to any loss of information from stage 1. Below we will show that the relative information in the two stages determines the efficiency of the UMVCUE compared to the stage 2 estimator, and that our estimator can include substantial information from stage 1. In these data, however, the closeness of the UMVCUE to the stage 2 estimates suggests that the stage 1 estimates are themselves close to their expected values after allowing for selection bias.

### BIAS AND MSE

To evaluate the bias and MSE of the proposed estimators, as defined in (12) and (13), we need to repeatedly simulate multiple effects from a genome scan. We took two approaches to doing this using the T1D and CD results as a model. In the first simulation (method *A*) we conditioned on detecting a fixed set of SNPs, so we consider genome scans that detect these and only these SNPs. This just involves sampling the log odds ratios from their normal distributions, truncated to allow for the *P*-value threshold. For each disease, we took the UMVCUE estimates and stage 2 allele frequencies from Tables I and II as fixed, and the same sample sizes as used in the original studies [Todd et al., 2007; Parkes et al., 2007]. Details of the sampling procedure are given in Appendix B. Here and subsequently our simulations use 10,000 replicates.

**Fig. 3:**
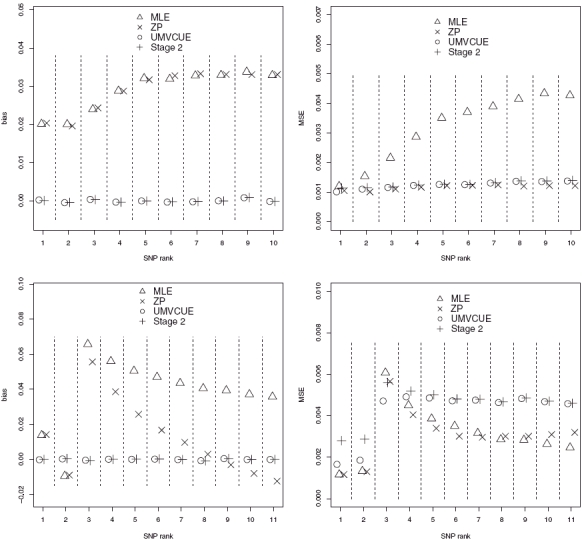
Top: Bias and MSE for T1D data. Bottom: bias and MSE for CD data. Numbers calculated using method A. TID, type-1 diabetes; CD, Crohn's disease.

We considered four estimators based on two-stage data: the unadjusted MLE, the corrected MLE of Zhong and Prenctice [2008] (ZP), our UMVCUE, and the estimate from stage 2 only. Figure 3 confirms that the MLE and ZP are biased, whereas the UMVCUE and stage 2 estimators are unbiased. Figure 3 also shows that the MSE of the UMVCUE is always less than that of the stage 2 estimator; the improvement is greater for the CD results, in which the stage 2 sample size was smaller than for the T1D study. The error of the UMVCUE can be either greater or less than that of the MLE; it seems to be more dependent on the sample size in stage 2, with greater error when stage 2 is small.

Notice that there is actually a downward bias in the MLE for the 2nd-ranked SNP in CD. This is because the power for the two highest ranking SNPs is so much higher than that of the others that these two SNPs are always the two highest ranking, but their power is roughly equal. The SNPs then form two subsets for which ranking bias operates within each subset but not between. This shows that any adjustment to estimates of multiple effects must take all the effects into account, as our UMVCUE does.

In the second simulation (method B) we allow for a genomewide set of SNPs and conditioned only on the number of SNPs detected. This was identified as a difficult computational problem [Ghosh et al., 2008], but if we assume that the standard errors of the stage 1 and 2 estimates are fixed and known, this reduces to a sampling problem with weights given by the marginal power of each SNP. We supposed the reported SNPs were representative of the true disease model, and assumed constant power of 1/3 to detect each reported SNP. We assumed an additional 300,000 independent null SNPs with minor allele frequency distributed uniformly on (0.05, 0.5). We then repeatedly sampled 10 or 11 (for the two disease models, respectively) SNPs passing the *P*-value threshold. Details of this procedure are also given in Appendix B.

**Fig. 4:**
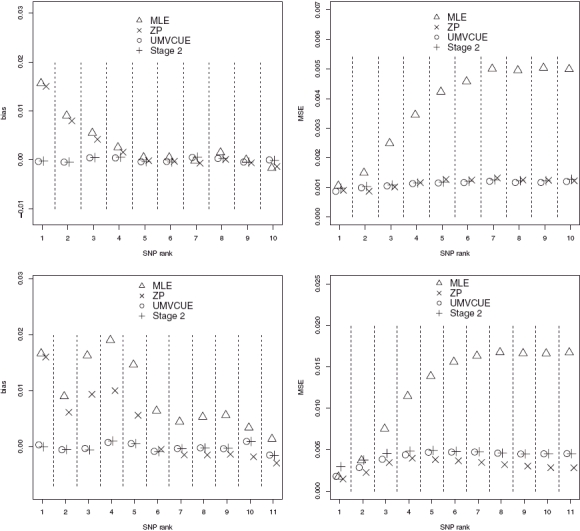
Top: Bias and MSE for T1D data. Bottom: bias and MSE for CD data. Numbers calculated using method B. TID, type-1 diabetes; CD, Crohn's disease.

Figure 4 shows a similar pattern to Figure 3, except that the downward bias for the 2nd-ranked SNP in CD is now a reduced upward bias, and the MSE of the MLE is now greater than for both the stage 2 and UMVCUE estimators. Apparently this is due to the inclusion of many null SNPs, for which the error in the naïve estimate is greater when such an SNP stochastically meets selection criteria in stage 1.

The ZP estimator performs well in terms of MSE, having in most cases the minimum of the four methods. Its bias is reduced compared to the unadjusted MLE, but not always by much, particularly for the top-ranking SNPs. The UMVCUE is unbiased as predicted, but its MSE tends to be higher than that of ZP, and sometimes of the unadjusted MLE too. This represents a bias-variance trade-off, but we know that the UMVCUE has minimum variance among unbiased estimators that condition on the stage 1 ranking. We consider this further in the section “Discussion”.

### BOOTSTRAP CONFIDENCE INTERVAL

We noted earlier that an analytic confidence interval is not known for the UMVCUE: the Rao-Blackwell theorem ensures but does not quantify the minimum variance property. However, since the UMVCUE is unbiased we can regard the MSE as a variance from which confidence intervals can be derived. If we simulate as above from the estimated effect sizes, then the variance gives a parametric bootstrap Wald-type interval subject to the assumptions and conditioning used in the simulation. Our first simulation approach has weaker assumptions about effect size and allele frequency, but stronger conditioning, while our second has stronger assumptions but weaker conditioning. An alternative simulation approach is to assume that all SNPs have equal frequency and therefore the same standard error [Bowden and Glimm, 2008], which we expect to give a worst case for MSE and therefore a conservative confidence interval, but one with weaker assumptions. Having estimated the MSE, a 95% confidence interval is estimated by 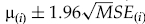. This assumes normality of the UMVCUE, which has been observed without proof [Bowden and Glimm, 2008]; but alternatively a quantile-based interval could easily be obtained from the bootstrap replicates to relax this assumption.

### REPLICATION SAMPLE SIZE

**Fig. 5:**
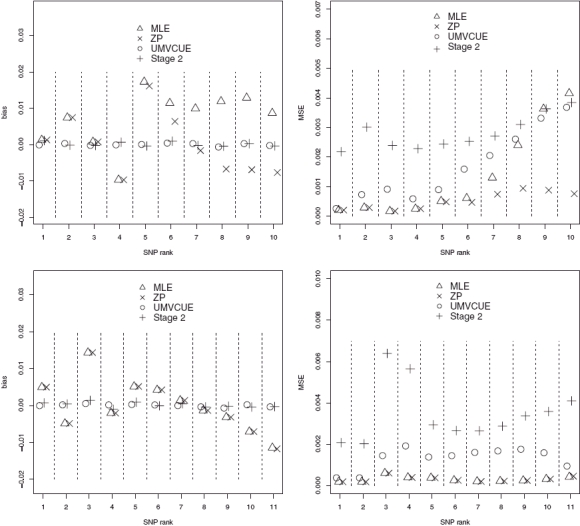
Top: Bias and MSE for T1D data. Bottom: bias and MSE for CD data. Numbers calculated using simulation method A, but with an assumed stage 1 of size 20,000 and stage 2 size 2,000. TID, type-1 diabetes; CD, Crohn's disease.

We have seen that the gain in efficiency of our UMVCUE over the stage 2 estimate depends on the sample size in stage 2, more precisely the relative size of stage 2 compared to stage 1. As stage 2 gets larger, our approach gives smaller gains in efficiency, although it is always more ef ficient. In a sense our examples from WTCCC were not ideal, because that study was designed as a proof of concept with a smaller sample size than might normally be attained, and the participating groups had access to a larger number of samples that became the de facto replication data even if they were not originally intended as such. While this may yet become a common model, we also considered a design with 20,000 cases and controls in stage 1 and 2,000 of each in stage 2, a possible scenario for future studies as genotyping costs decrease and samples are shared among consortia, such as for meta-analysis. Using simulation method A, conditioning on detecting a fixed set of SNPs, Figure 5 shows that there is a more substantial gain in efficiency from our approach, particularly for the top-ranking SNPs.

**Fig. 6:**
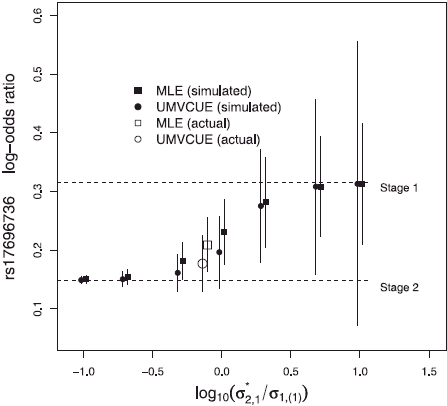
Point estimates and bootstrap confidence intervals for the MLE and UMVCUE for rs17696736, varying the stage 2 standard error relative to stage 1. The point estimates and bootstrap confidence intervals are calculated using method A.

The relative sizes of stages 1 and 2 also affect the value of the UMVCUE 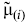, most importantly through the role of the standard errors σ_1,(*i*)_, and σ_2,*i*_ but also through σ_1,(*i–1)*_ and σ_1,(*i*+1)_. Figure 6 illustrates this point for estimates of the top-ranking stage 1 SNP, rs17696736, in the T1D data [Todd et al., 2007]. Recall that there is a substantial difference between the stage 1 and 2 estimates for this SNP, but the UMVCUE is very close to the stage 2 estimate. In the original study, the ratio of the stage 2 estimate's standard error over the stage 1 standard error, σ_2,1_/σ_1,(1)_, was approximately 0.75. In a simulation study we replaced the actual value of σ_2,1_ by 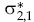, and varied this in the range (0.1σ_1,(1)_, 10σ_1,(1)_) to demonstrate how the MLE and UMVCUE estimates change. At each value of 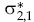 the variance of the UMVCUE and MLE were evaluated by simulation as before. When the stage 2 replication study is large relative to stage 1, it dominates the value of both estimators. When the opposite is true they both tend toward to the stage 1 estimate, but the variance of the UMVCUE increases in magnitude. This reflects the fact the UMVCUE remains unbiased: even if it is close to the MLE, the confidence interval covers values of μ_(1)_ close to the stage 2 estimate. By the same token, if the true value of μ_(1)_ is close to the stage 2 estimate, the larger variance of the UMVCUE allows it to attain values close to the MLE without incurring bias. Note that if μ_(1)_ is indeed close to the stage 2 estimate, then unbiasedness implies that the UMVCUE will sometimes be less than the true value, so will not be close to the MLE for every sample. Although the UMVCUE and MLE can become arbitrarily close as stage 1 gets larger, this will not be true for every sample drawn at a fixed and finite stage 1 size.

### *P*-VALUE THRESHOLD

Because our adjustment to the ordinary MLE depends only on the two flanking stage 1 estimates, any *P*-value threshold affects only 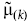, the estimate for the least significant SNP taken forward from stage 1. In fact, adjusting for the values of the flanking estimates automatically ensures adjustment for selection by significance and rank. A reassuring consequence is that all but one of our estimates are robust to mis-specification of the *P*-value threshold.

In Table III we show the estimated effect of the lowest-ranking SNPs from the T1D and CD data, for a series of assumed *P*-value thresholds. It is clear that the estimate is fairly robust to this threshold so long as it is not grossly mis-specified.

**TABLE III:** Estimated effects of the lowest-ranking SNPs for various assumed *P*-value thresholds

	5×10^−5^	10^−4^	10^−3^	0.01	0.05
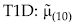	1.00144	1.00545	1.0226	1.0442	1.0620
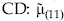	1.438	1.449	1.480	1.491	1.492

SNP, single-nucleotide polymorphism.

## DISCUSSION

Correcting for selection bias in genome scans is problematical because no unbiased estimator exists for the scan data alone. We have proposed a two-stage estimator that is unbiased while including the data from a genome scan, and moreover accounts for selection bias by both significance and rank. Our approach explicitly allows for multiple associations arising in a scan, which is important because of the different degrees of ranking bias induced, and also the possibility, as we have seen, of negative bias in some circumstances. We have shown that this estimator gives improved precision compared to estimation based only on replication data, with the greatest improvements when the replication sample size is smaller than the initial scan.

We argue that in many of the applications for which estimation is needed, associations should already have been replicated so that the two-stage design will usually be available. For example, it seems premature to estimate the total attributable risk for the detected loci until those loci have been confirmed. Of course, we are not advocating collection of a second sample only for unbiased estimation, and estimation from the scan data alone will sometimes be necessary, such as in setting the sample size for a replication. One-stage bias-reduced estimators have been proposed [Ghosh et al., 2008], which undoubtedly have utility when their mean square error is small. Our intention here is to explore what can best be done when two-stage data *are* available. All approaches, including ours, become much more accurate than those based only on the first-stage data. Indeed, we found that the bias and mean squared error of the method of Ghosh et al. [2008] for the simulation studies in the section “Results” were in general considerably larger than the two-stage methods (data not shown).

Nevertheless, if only initial scan data are available, we note that our approach can be incorporated into a single-stage analysis. Sun and Bull [2005] proposed a bootstrap estimate of the ranking bias by randomly splitting the sample into discovery and validation subsets [see also Yu et al., 2007]. The difference between estimates in the two subsets gives an estimate of the bias, but we can also use the difference between the discovery estimate and our combined estimate from the two subsets. Our approach can therefore in theory be combined with that of Sun and Bull [2005] to obtain a more accurate bootstrap correction in a single-stage design. This appears to be an interesting topic for further work.

Our focus in this article has been on unbiased point estimation of SNP effects. However, in the section “Replication Sample Size” we saw that while the two-stage UMVCUE for a top-ranking stage 1 SNP tended toward the biased stage 1 estimate as the stage 2 precision was set close to zero, its bootstrapped confidence interval width increased to keep the estimate nominally unbiased. This suggests the possibility that when only heavily selected stage 1 data are available, by postulating an unbiased estimate with a large variance (in the manner of a vague prior), the resulting UMVCUE confidence interval could also be used for hypothesis testing, to help distinguish null effects from true effects of small magnitude. It would be interesting to assess the performance of this ad hoc proposal relative to other more established multiple testing strategies.

We considered a two-stage design consisting of a genome scan and a replication study, but two-stage genome scans can also be performed [Satagopan et al., 2004]. We did not consider this design because selection is likely to occur after the second stage as well as the first. That is, not all the markers selected from the first stage are expected to be true positives, and only the markers that remain significant after the second stage are deemed to be of further interest. In contrast, unsuccessful replications are still reported following a single-stage scan [Todd et al., 2007] and such markers may remain targets for replication in the future. Our methods originate from the clinical trials literature [Stallard and Todd, 2003; Bowden and Glimm, 2008] in which competing treatments are compared at interim stage and the most promising ones then taken forward. This design is similar to the genome scan situation although the end motives are sometimes different. For example, in such a clinical trial it is important to select the “correct” treatments at interim, that is we want the top-ranking treatments at interim to be those with the truly strongest effects, so that resources are then directed to the right targets. In genomewide association scans it is more important to detect a large number of true effects, accepting that power is incomplete and that some effects of similar strength will not be detected. Again, in a clinical trial the total sample size is a limiting aspect, giving a trade-off between selecting the right treatments (with a large stage 1) and accurately estimating their effects (with a large stage 2). To date this has not been a critical issue in genome scans as the primary aim is to ensure a sufficiently large stage 1 to detect many true effects, and replication has generally not been an integrated element in the design. This may change with time, leading to trade-offs similar to those in two-stage scans [Satagopan et al., 2004], but in which the false-positive rate in stage 1 is controlled more strictly.

Our estimator has the minimum variance among unbiased estimators that condition on the ordering in stage 1, but this holds only marginally for each estimate and not jointly. That is, each of 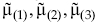,…, can be regarded as having minimum variance when considered singly, but when considering the vector of effects μ there may be multivariate estimators with smaller variance. This is because we consider only the two flanking estimates for each effect, whereas there is information in the other estimates that could potentially be taken into account. A jointly minimum variance estimator conditioning on the same ordering remains an open problem.

We give a formula based on ranking by *P*-values from a Wald test, but the current test of choice is the Cochran- Armitage test of trend, which is the score test from a logistic regression model [Sasieni, 1997]. The Wald test gives a closed form expression in terms of estimated odds ratios and standard errors, and in large samples the score and Wald tests are very close. Nevertheless our approach can be applied to a ranking based on any function of suitable summary statistics, including score tests and also Bayes factors [WTCCC, 2007]. Let *T* be a statistic on which SNPs are ranked, and suppose it can be computed from an estimate of the odds ratio *X* and some nuisance parameters θ. Write *T = T*(*X*; θ) and let *T*^−1^(*x*; θ) be the solution of *T*(*X*; θ) = *x*. Then equation (11) becomes


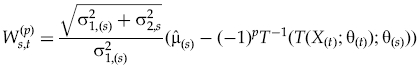
14

We applied this approach to Pearson χ^2^ tests from the 2 × 2 allelic contingency tables inferred from the reported summaries [Todd et al., 2007; Parkes et al., 2007]. Here the nuisance parameters θ were the allele frequency, sample size, and case/control ratio. The results were almost identical to those obtained from the Wald tests (data not shown).

Our results show that while our estimator is unbiased, it can have greater mean square error than the two alternatives we considered for this design: the unadjusted standard combined estimate, and the significance-corrected estimator of Zhong and Prenctice [2008]. Although those competitors are biased, the magnitude of the bias is often small, and perhaps acceptable in view of their smaller MSE. This bias-variance trade-off is a common problem in choosing an estimator, but we think that unbiased ness is an important goal in genomewide association studies for two main reasons. Firstly, the sample sizes are so large that the MSE is small in absolute terms for the UMVCUE also, and when the expected error is small it is tempting to take the estimate at face value. Secondly, as the effect sizes in genome scans are small, the bias in estimation will influence decisions on whether associations are worth following up: essentially, the bias will influence the false discovery rate, and it seems worthwhile to reduce this possibility.

We have assumed that all effect estimates are independent. This is approximately true for the studied examples since replication was attempted for one SNP in each genomic region, with two exceptions (5p13 and 5q33 in CD) in which the replicated SNPs were in weak linkage disequilibrium (LD). However, each SNP was selected for being the most significant in its local region, so that some ranking bias is present in the stage 1 estimates that is not accounted for by our approach.We do not expect this to be a serious problem since the ranking bias decreases as LD increases, so that when our failure to account for LD is most acute, the resulting bias is minimal. Nevertheless, a precise unbiased estimator that takes into account known or estimated LD structure is an open problem for further work.

Finally we note that our approach extends trivially to a meta-analysis of several studies following an initial scan, by treating the combined estimate from the replication studies as our stage 2 and then combining it with the initial scan to obtain an overall unbiased estimate. R code to implement the method proposed in this article is available from the authors on request.

## APPENDIX A RANKING BY TWO-SIDED P-VALUE AND THRESHOLD

For *i* = 2,…, *k* – 1 we note that

implies

Also, the fact that

implies

Putting these two bounds together, and also noting that

(A1)

For *i* = 1 we replace σ_1,*i*_|*X*_*i*–1_|/σ_1,*i*–1_ with ∞ and for *i* = *k* we replace σ_1,*i*_|*X*_*i*+1_|/σ_1,*i*+1_ with σ_1,*i*_Φ^−1^(1 – *p*_crit_/2).

Modifying the proof to take into account the new thresholds for *X_i_* we must evaluate the integral

(A2)

to derive . Calculating  involves a similar two-component integral, and leaves the expression in equation (10).

## APPENDIX B SIMULATION OF SIGNIFICANT EFFECT SIZES

We first simulate from a fixed effect size, conditional on significance of a two-sided Wald test at *P*<*p*_crit_. Let *q* = Φ^−1^(1 – *p*_crit_/2. The left- and right-sided power to detect effect size μ with variance σ^2^ is respectively

(B1)

(B2)

With probability π_l_/(π_l_ + π_r_) we sample from the left tail of the distribution of μ according to

(B3)

where *U*_l_ is a Uniform(0, π_l_) deviate, and with probability π_r_/(π_l_ + π_r_) we sample from the right tail according to

(B4)

where *U*_r_ is a Uniform(0, π_r_) deviate. This is sufficient to simulate sample estimates conditional on detecting a fixed set of SNPs. To allow for a genomewide scan and condition only on the number of SNPs detected, we define effect sizes and standard errors for all SNPs in the scan and sample without replacement using the total power π_l_ + π_r_ as the weight (we used the sample ( ) function in R). The sampled effects are then input as fixed effects into the above scheme.
